# Lower Urinary Tract Dysfunction in Myasthenia Gravis: A Case Report and Literature Review

**DOI:** 10.7759/cureus.72553

**Published:** 2024-10-28

**Authors:** Patrícia Pereira, Manuela Mira Coelho, Sónia Tizón, Sara Freixo

**Affiliations:** 1 Physical Medicine and Rehabilitation, Hospital de Braga, Braga, PRT

**Keywords:** lower urinary tract symptoms, myasthenia gravis (mg), stress urinary incontinence, urge urinary incontinence, urodynamic

## Abstract

Lower urinary tract dysfunction often occurs after various neurological disorders and significantly impacts patients' quality of life. Studies on myasthenia gravis (MG) are scarce, and most of them are case reports associated with symptoms such as overactive bladder, incontinence, and urinary retention.

The authors present the case of a 68-year-old female patient with a myasthenia gravis diagnosis, followed by Urogynecology Physical Medicine and Rehabilitation for stress urinary incontinence. A urodynamic study performed demonstrated intrinsic sphincter dysfunction with detrusor hypocontractility. She began functional rehabilitation of the pelvic floor without improvement, so she was advised to consider a possible surgical approach.

This case report highlights the connection between MG and lower urinary tract dysfunction, underscoring the necessity for increased awareness and customized management strategies for affected patients.

## Introduction

Myasthenia gravis (MG) is a rare autoimmune disease of the neuromuscular junction caused by the development of antibodies against proteins in the postsynaptic membrane of the neuromuscular junction, which could be acetylcholine (Ach) receptors or receptor-associated proteins. A disruption in neuromuscular transmission results in fluctuating fatigue and limb and bulbar muscle weakness. The disease tends to have a bimodal distribution to the age and sex predominance of onset, with an early peak in the second and third decades (female predominance) and a late peak in the sixth to eighth decades (male predominance). MG can also be classified based on clinical form, ocular or generalized, or by the presence of specific autoantibodies targeting receptors at the neuromuscular junction. The prognosis of MG varies by symptom severity and response to treatment, patients with severe or refractory symptoms are at higher risk for complications compared with those with mild or nonbulbar symptoms.

The diagnosis of MG may be confirmed by the presence of autoantibodies against the acetylcholine receptors or other muscle receptor-associated proteins (muscle-specific tyrosine kinase (MuSK) or low-density lipoprotein receptor-related protein 4 (LRP4)). In approximately 70% of the patients with MG, the antibodies present are against nicotinic ACh receptors found in the post-junctional membrane of striated muscles [1].

The symptomatic treatment of MG with cholinesterase inhibitors (Pyridostigmine) retard recycling the acetylcholine at the neuromuscular junction enabling a more extended interaction with receptors of Ach. Nevertheless, inhibition of cholinesterase in other tissues leads to side effects of the muscarinic ACh receptor hyperactivation in the autonomic nervous system, initially in smooth muscles, including the bladder and bowel.

Micturition is achieved through complex neurological mechanisms involving somatic, autonomic, and central components. The parasympathetic nervous system stimulates muscarinic receptors in the smooth muscle bladder wall triggered by urethral relaxation. Neuronal nicotinic acetylcholine receptors control bladder function, mediating fast synaptic transmission between preganglionic and postganglionic bladder neurons [2].

Lower urinary tract dysfunction often occurs after various neurological disorders and significantly impacts a patient's quality of life. In MG, studies are scarce, and most cases report overactive bladder, incontinence, and urinary retention.

This article was previously presented as a poster at the XV APNUG Congress on September 27, 2024.

## Case presentation

The authors present the case of a 68-year-old female patient, functionally independent for activities of daily living, following a Urogynecology consultation at the Physical Medicine and Rehabilitation (PMR) department due to stress urinary incontinence. She had a history of a thymoma submitted to thyroidectomy in 2015 and a subsequent diagnosis of myasthenia gravis in 2017.

She had electromyography with right facial nerve decrement on repetitive nerve stimulation and Ach receptor antibody positive. On neurological exam, she presented with scarce bilateral palpebral ptosis with fatigability without other motor or sensitive limitations. She has been medicated with azathioprine 100 mg daily, sertraline 100 mg daily, and prednisolone 2.5 mg on alternate days.

The patient reports experiencing symptoms of stress incontinence and sometimes continuous leakage after MG diagnosis in 2017, requiring the use of four to five daily incontinence pads. She mentioned that these symptoms severely impacted her quality of life. On objective examination, she demonstrated a positive stress test with an empty bladder, muscle strength grade 1 on the modified Oxford scale, decreased anal tone, and decreased anal sphincter contractility.

**Figure 1:**
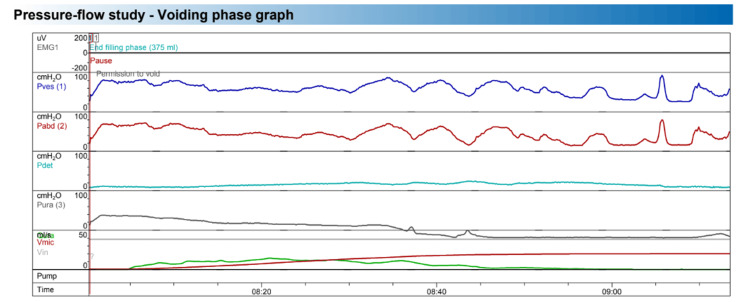
Pressure-flow study of the patient during the voiding phase The voiding phase demonstrated abdominal contraction without detrusor contraction.

She underwent a urodynamic study that revealed, on cystometry, an abdominal leak point pressure (ALPP) of 57 cmH_2_O, a urethral pressure profile demonstrated a maximum urethral closing pressure (MUCP) and a urethral functional length decreased of 25 cmH_2_O and 18 mm, respectively. Uroflowmetry evidenced a normal uroflow pattern with no post-void residual volume; pressure-flow study demonstrated absence of detrusor overactivity, average maximal cystometric capacity (MCC) of 380 ml, normal compliance of 90 ml/cmH2O, voiding phase demonstrated abdominal contraction without detrusor contraction, with non-obstructive flow and zero residual volume (Figure 1). This data suggests intrinsic sphincter dysfunction with an underactive detrusor.

The patient began functional rehabilitation of the pelvic floor without symptom improvement, so she was referred for evaluation in a multidisciplinary consultation with urology to consider a possible surgical approach.

## Discussion

A literature review was made of the PubMed database and eight case reports and three comparative studies published in 2021 were found. Tateno F et al. [2], Akan O et al. [3], and Donskov AO et al. [4] demonstrated that MG patients have a statistically significant increase in overactive bladder symptoms compared to healthy controls. Also, Tateno F et al. reported that the quality of life of MG patients was severely affected by lower urinary tract symptoms, therefore, bladder dysfunction must become an essential therapeutic target in these patients. Akan O et al. studied 36 patients with MG and similarly demonstrated that patients with MG had high postvoid residual urine volume, particularly in patients with late-onset MG [3]. MG seronegative patients seemed to have a prolonged duration of disease and urinary disturbances. The limitation of these three studies was the absence of urodynamics, as they only included questionnaires and uroflowmetry and ultrasonography data in the Akan O et al. study.

Detrusor hyperactivity is generally attributed to lesions of the first-order neuron but can theoretically originate from lesions of the second neuron associated with neuronal reorganization and consequent denervation. However, detrusor hyperactivity can also occur in MG and is associated with pyridostigmine medication, commonly prescribed to improve muscle weakness in these patients by stimulating bladder muscarinic receptors. Nocturia might derive from nocturnal polyuria since many patients were taking oral prednisolone, and polyuria induced by cortisol has been described [5].

While often used for treating overactive bladder, anticholinergics are contraindicated for MG. Intradetrusor onabotulinumtoxinA should be cautiously administered to patients with MG, though individuals with underlying neuromuscular conditions can develop more generalized weakness. Therefore, to treat overactive bladder in MG, serotonergic drugs (serotonin-noradrenaline reuptake inhibitors), b3-adrenergic receptor agonist drugs (Mirabegron), and neuromodulation may be the best options. Recently, published in 2024, Kwegyir-Aggrey A et al. reported a case of a 71-year-old patient who initially presented with urinary incontinence and later developed fatigable ptosis and diplopia [6]. Investigation revealed positive MusK antibodies and an MG diagnosis was made, with poor response to pyridostigmine and improvement of symptoms, including urinary incontinence, with two doses of Rituximab. Antoniou A successfully reported the successful treatment of detrusor over-activity in a young patient with myasthenia gravis using neuromodulation as pretibial nerve stimulation [7]. Previously, Pannek J and Grigoleit U presented a case of a child with congenital MG who was successfully treated with suprapubic functional electrical stimulation for overactive bladder symptoms and nocturnal enuresis [8].

On the other hand, symptoms of stress urinary incontinence in MG may be associated with urethral hypermobility due to support muscle failure or even opening of the urethral neck with decreased urethral closing pressure. When stress urinary incontinence is proven by examination and tests, functional rehabilitation of the pelvic floor is the first treatment option, with good results in failure of urethral support but better in intrinsic sphincter deficiency. Despite the risk of rejection in case of detrusor hypocontractility or worsening detrusor overactivity, surgery for counteracting pelvic floor descent is the treatment choice when conservative treatment fails.

Studies also report urinary emptying dysfunction, namely, detrusor hypocontractility. Most of the case reports involved urinary incontinence in males, which developed after transurethral resection of the prostate, but some of them were not associated and may originate from peripheral nerve injuries or detrusor injuries [9-13]. Theoretically, MG may co-occur with autoimmune autonomic ganglionopathy, inhibiting the detrusor through ganglionic blockade of the nicotinic ACh receptor to generate detrusor hypocontractility. Autonomic dysfunction in patients with MG might indicate a unique subset with a worse prognosis. Marouani I et al. and Matsui M et al. reported two cases of seronegative MG associated with bladder dysfunction and detrusor areflexia [9,13]. Finally, the role of sphincter innervation on voiding dysfunction in MG is controversial, given that it has triple somatic, adrenergic, and cholinergic innervation. Comorbid neuropathy in MG may be associated with sphincter obstruction due to partial somatic and sympathetic denervation.

Table 1 lists all the related studies found during the literature review. 

**Table 1:** Case reports reviewed from the PubMed database

	Sex	Age	Disease	Symptoms	Urodynamic study	Treatment
Kwegyir-Aggrey A et al. (2024) [6]	Female	71 years old	MuSK-Associated Myasthenia Gravis	Urinary incontinence non-specific	-	Rituximab
Antoniou A (2016) [7]	Female	24 years old	MG	Mixed urinary incontinence, urgency predominance	Unprovoked detrusor contractions with urgency	Neuromodulation with pretibial nerve stimulation 12 weeks
Pannek Jurge and Grigoleit Ute (2008) [8]	Male	6 years old	Congenital MG	Nocturnal enuresis and urgency incontinence daily	-	Functional electrical stimulation applied via suprapubic surface electrodes
Marouani I et al. (2012) [9]	Female	13 years old	Seronegative MG	Dysuria and urine retention	Detrusor areflexia and urethral sphincter hypertonia	Clean intermittent catheterization
Kaya C and Karaman MI (2005) [10]	Female	61 years old	MG	Pollakiuria, feeling of incomplete bladder emptying, and recurrent urinary tract infection	Detrusor areflexia	Clean intermittent catheterization
Christmas TJ et al. (1990) [11]	Female	35 years old	MG	Difficulty in voiding, pollakiuria, and feeling of incomplete bladder emptying	Detrusor areflexia	-
Sandler PM et al. (1998) [12]	Female	39 years old	MG	Urinary retention	Detrusor areflexia	Clean intermittent catheterization
Matsui M et al. (1995) [13]	Female	20 years old	Seronegative MG	Urinary retention	-	Urinary catheterization

## Conclusions

This case report emphasizes the connection between myasthenia gravis and lower urinary tract dysfunction, highlighting the need for tailored management strategies. It details significant urinary symptoms in an MG patient, such as stress urinary incontinence and underactive detrusor function, aligning with existing literature on MG-related urinary symptoms. Managing these symptoms is complicated by the contraindications of common medications like anticholinergics, which may worsen MG symptoms. A multidisciplinary approach involving urologists, neurologists, and rehabilitation specialists is essential for effective treatment. Future research should involve larger studies and urodynamic assessments to better understand urinary dysfunction in MG and explore alternative therapies to improve patient care and quality of life.
